# Commentary: Industry collaboration: A call for ‘industry literacy’ – a commentary on Bourgaize et al. (2025)

**DOI:** 10.1111/camh.70017

**Published:** 2025-07-27

**Authors:** Bernadka Dubicka, Richard Graham, Harriet Over, Lewis W. Paton, Thees Spreckelsen, Paul A. Tiffin, Heather Wardle, David Zendle

**Affiliations:** ^1^ Hull York Medical School University of York York UK; ^2^ Department of Health Sciences University of York York UK; ^3^ Department of Psychology University of York York UK; ^4^ School of Social and Political Sciences and School of Health and Well‐Being University of Glasgow Glasgow UK; ^5^ School of Social and Political Sciences University of Glasgow Glasgow UK

## Abstract

In response to the article by Bourgaize et al. (*Child and Adolescent Mental Health*, 2025) on academic collaborations with technology companies, we argue that we need to move beyond guidance for individual researchers; instead, there is an urgent need to develop a research infrastructure, to manage the risks of collaborating with corporations whose profits rely on the very products under investigation. Institutional transparency is essential as well as consideration of the wider ecosystem regarding conflicts of interest. Much can be learned from historical examples of ‘corporate playbook’ techniques, such as the gambling and tobacco industries. Specialist ethical oversight is urgently needed, which considers broader questions around commercial influence, and minimum open science standards should be mandated by research institutions, in order to preserve public trust in science. An overarching national center of expertise is needed to develop guidance, together with legislation to enforce data sharing for independent research. Lastly, we suggest detailed questions should be asked about who may have the most to lose and the most to gain from a collaboration; academics should equip themselves not just with digital literacy, but also with ‘industry literacy’ to navigate this complex relationship.

We welcome the article by Bourgaize et al. (2025) for raising a vital, but rarely discussed, issue for academics working in the digital age. Technology has advanced rapidly in recent years, whilst the academic evidence base continues to be left behind. The UK Royal College of Psychiatrists called for government action in 2020, compelling data sharing (RCPsych, 2020); however, subsequent progress has been limited, and a lack of access to technology companies' data for independent research remains a major barrier.

How can we truly understand the impact of the digital world on children and young people (CYP) without access to the real‐time data held by these companies? Given the limited ability of individuals to self‐report digital use, collaboration with technology companies to access the data and insight they generate appears to be an appealing way forward. However, whilst such relationships may afford unprecedented insights into the digital world, they may also create powerful opportunities for industrial stakeholders to influence research ecosystems.

In this commentary, we build on Bourgaize et al's article to reflect on four key areas for guidance associated with collaborating with industry. We then argue for the need to go beyond guidance for individual researchers; instead, we propose the development of stable research infrastructure, which manages the risks for individual researchers that occur when collaborating with corporations whose profits rely on the very products under investigation.

## Guidance domain 1: Institutional transparency and conflicts of interest

As Bourgaize et al. note, ‘collaboration is already widely promoted by universities and funders’. Academic systems of incentivization for career progression may encourage researchers to collaborate with commercial sectors, especially where funding is involved, due to the scarcity of other available funding. However, after such agreements are brokered, the perception of undue influence by corporations upon an individual's research may be difficult to shift. Early career researchers may find they have to continually justify their funding arrangement and the independence of their results: decisions made during the early stages of their career can substantially affect their future opportunities.

Questions around these collaborations are rarely discussed within universities, funders, or ethics boards. A deeper understanding of the wide range of mechanisms by which commercial entities may influence research is nascent outside of isolated subfields (e.g., tobacco research).

Just as an individual's reputation and ability to generate evidence may be compromised by industry involvement, so too may larger institutions, bodies, and even entire subfields. The Lancet Commission on Gambling (Wardle et al., 2024) describes the ‘corporate playbook’ of the gambling industry, such as shaping the evidence by funding academic projects, controlling access to data, discrediting critical findings, and formulating research agendas that align with their preferences. Similarly, junk food manufacturers have aligned youth health research with physical activity rather than diet (Kmietowicz, 2015). A similar trend may already be emerging within technology research, with emphasis in the literature drawing toward ‘safe’ technology use rather than other, potentially corporate‐unfriendly options, such as significant reductions in screen time.

Public trust in science is fragile. A survey of 68 countries concluded that scientists wishing to gain public trust needed to be more transparent about funding and data sources, as well as invest more effort into communicating with the public (Cologna et al., 2025). Collaborations that appear captured by commercial interests jeopardize not only individual reputations but also confidence in academic institutions and policy processes. Given the context above, guidance given to individual researchers must cover both the safeguarding of their own individual interests, as well as these broader ecosystem issues.

## Guidance domain 2: Ethical oversight and specialist input

As Bourgaize et al. note, institutional ethical approval may act as an ‘existing safeguard’ for the research process. However, many ethics boards are unfamiliar with the complex issues that arise when collaboration with industry occurs and are hence ill‐equipped to provide the specific level of oversight and expertise that is required when managing such research. Current processes arguably tend to focus on participant protection and rarely deeply interrogate broader questions of commercial influence or interference unless a specific domain (e.g., tobacco) is invoked.

The due diligence checklist proposed by Bourgaize et al. forms a starting point for the development of such an inspection. However, it heavily emphasizes the value of voluntary industrial promises such as corporate Patient and Public Involvement and Engagement (PPIE). It is not clear that such due diligence checks are credible. For example, a company may have signed up to best practice guidance and involved PPIE, but this may simply be a tokenistic public relations exercise without meaningful engagement.

Guidance to researchers must thus incorporate additional, domain‐specific frameworks in order to help them understand the extent to which industry initiatives and associated organizations (e.g., Industry Social Aspect Organisations, which ostensibly prioritize social and environmental responsibility) can truly ensure ethical and safe practice.

In a European academic position statement, the All European Research Alliance (ALLEA, 2025) states that there is no ‘one‐size‐fits‐all’ solution or algorithm that can tell research institutions which collaborations may be ethically justifiable. The authors suggest that case‐by‐case analysis is needed. This includes assessing whether the vision, mission, and values of the academic institution can be contradicted by the collaboration. That is, would it threaten undermining science and reputations and whether there is an impact on academic freedom and open science? The authors suggest that if any of these keystones are encroached upon, research institutions violate their responsibility to deliver reliable research findings and/or to uphold public trust in science by being untrustworthy. The authors state that the ‘risks of tainting an institution's reputation is greater if it is connected to areas that are highly polarised in public debate: even when not directly violating the principles of research integrity, collaborations may contribute to reducing public trust in science’. Lastly, they discuss the need to consider the relative distribution of benefits and burdens, including immaterial benefits such as the positive reputational effects of a company's collaboration with an established research institution.

ALLEA make the recommendation that independent committees should evaluate specific cases, which could be an ethics review board that has developed the relevant expertise: guidance is needed for researchers to allow them to both develop this expertise and evaluate whether their local review structures have obtained such expertise.

## Guidance domain 3: Transparency and due diligence

Bourgaize et al. mention the substantial guidance developed for and by researchers working in the context of industries such as tobacco and pharmaceuticals. These contexts provide excellent historical examples of why independent research can be problematic due to data transparency issues where there are competing and overriding commercial interests. For example, evidence suggests that much of the original research conducted into the effectiveness of SSRI antidepressants deliberately avoided recording common and serious side effects. These included increased suicidality in adolescents (Jureidini et al., 2004).

Understanding data quality and completeness is compounded by data sharing agreements, which can create the ability for industry to select which data they are willing to share with researchers. This is further compounded by situations (e.g., Pearson et al. 2024) where researchers have been unable to replicate studies using industry data. Unless tightly governed, negative findings that are misaligned with commercial interests may be suppressed. Alternatively, they may never be generated due to data pipeline interruptions: researchers may have limited means of knowing whether companies are being transparent with data that they share or whether data has been selectively withheld. History may therefore warn us that guidance must integrate enforceable ways to scrutinize access and verify transparency. Without this, collaboration may entrench rather than mitigate bias.

Transparency and due diligence extend to the whole research life cycle, from study planning to dissemination. Open science practices include well established tools to reduce research biases, such as publicly available study protocols and pre‐registered plans of analysis prior to the start of the project, through to code and data sharing at the conclusion of the research. Some aspects of open science, such as data sharing, are more complex and often not legally possible, particularly when collaborating with industry. Nevertheless, institutional frameworks and journals should clearly mandate minimum open science standards that should be met. We would suggest, at a minimum, that publicly available preregistration of an analysis plan is required to reduce the risks of the influence of commercial interests. For the individual researchers, this would also provide an opportunity to publicly define the role industry partners will play in a study. For academic institutions that are increasingly sensitive to research integrity, protocols and pre‐analysis plans could become an element of an integrity review or requirement to approve collaborations. For journals and the public, they offer both a basic safeguard against biases and a means to achieve accountability, especially where conflict of interest statements often only declare potential conflicts, yet do not require highlighting of potential safeguards against bias and for independence.

## Guidance domain 4: Managing subtle commercial interests and risks

Bourgaize et al state that the risks of collaboration are often hard to identify. This points to a specific area in which researchers require substantial guidance: identifying, triaging, and dealing with subtle forms of industry bias. It is straightforward to understand how accepting money from an industry body might lead to biases in the research process. However, there are different levels of potential collaboration with industry bodies. Each layer in any collaborative hierarchy bears with it the risk of compromise and conflict of interest. Examples of such collaboration levels are:

1. Receiving financial funding for research.
2. Receiving privilege access to data or other substantial ‘in kind’ resources such as expertise via industry staff time.
3. Being offered access to other resources for free, or at a discounted cost, such as apps, or tools.
4. Co‐hosting or speaking at academic events where some or all of the funding is from industry.
5. Co‐authoring published research reports or opinion pieces.

This is clearly not an exhaustive list. However, aside from funding, not all of these activities would be routinely declared, but each has potential risks for research independence, trust, and transparency, which researchers need to carefully consider and weigh. Researchers should always be attuned to how any collaborations with commercial entities serve the interest of those entities and consider whether this is aligned with public interest. Researchers require guidance regarding when, why, and how these forms of collaboration might impact their credibility and findings.

## Data infrastructure—A way forward

Much more work needs to be done regarding guidance for researchers, starting with institutional reviews and principles that align with their values, public trust, and academic freedom. The foundation for this depends upon a greater level of digital literacy as much as data science, and a greater understanding of the likely business model of any company. We would suggest that researchers undertake a risk assessment for any potential bias or distortion of a proposed methodology, and additional important questions for researchers would be:

- Under what circumstances could you consider a collaboration?
- What might you lose in this collaboration and what does the company stand to gain?
- Is there alignment between private and public interest?
- Can you trust the company? How will you know? (In the UK, the researcher may want to check on the companies' transparency reports for the Digital Services Act, evidence of compliance, and consider liaising with the regulators).

If there is doubt about this or interests are not aligned, we would advise caution and perhaps consider *interactions* rather than collaborations.

Finally, this debate highlights the urgent need for an overarching national center of expertise to develop such guidance, disseminate expertise, and give independent advice. However, the issue of data transparency and sharing will also need enforcement by legislation. Ofcom, the UK regulator, launched a Call for Evidence in October 2024 on Researcher Access to Data, as it is enshrined within the UK Online Safety Act:

‘The Act requires us to consider how independent researchers can access information from regulated online services to conduct research into online safety matters’.

In addition: ‘We must also consider “legal and other issues” that might constrain the sharing of information for these research purposes in our report’.

Ofcom is due to publish its report on Researcher Access in 2025, which may provide guidance on these issues; however, researchers and academic institutions should be learning from historical examples, and urgently consider who may have most to gain from collaborations with industry, and who may have most to lose; in other words, there is an urgent need not only for digital literacy, but also for researchers to acquire an ‘industrial literacy’.

## Conflict of interest statement

BD is editor in chief of CAMH; Royal College of Psychiatrists (RCPsych) co‐lead on digital safety. RG is an independent Child & Adolescent Psychiatrist in private practice and Online Harms Lead for the Child & Adolescent Faculty, RCPsych. As a member of Ofcom's Making Sense of Media Advisory Panel, he participated in meetings that included industry representatives. RG has undertaken paid consultancy to industry and charities: Member of Yubo's Trust and Safety Board; Praesidio Safeguarding on a project funded by TikTok; a digital health company, TalkLife; a digital mental health charity, stem4. LWP collaborated with Anathem on a research grant application for the Wellcome Trust Accelerator scheme. TS previously worked as a researcher on an industry‐funded study on the benefits of Omega‐3 fatty acids and has undertaken paid statistical analysis work for the International Catholic Relief Services, as well as providing paid training for the Department of Work and Pensions. PAT collaborated with Anathem on a research grant application for the Wellcome Trust Accelerator scheme; has done consultancy for Wellcome and for Work Psychology Group on behalf of the University of York, and consultancy for SAMMI Select. HW declares reimbursement for travel from the Gambling Regulators European Forum, and consultancy fees from the Institute of Public Health, Ireland, National Institute for Social and Economic Research, and Middlesbrough Council. She declares occasional attendance at events where the gambling industry is present, as required by the Gambling Commission, which funds the Gambling Survey for Great Britain. Her attendance at these events is independently funded and does not constitute a collaboration or partnership with the gambling industry. DZ has previously been involved in brokering a data sharing agreement with Unity Technologies, a stakeholder from the video games sector. He is interim chair of the Advisory Board for Safer Gambling, an independent expert body whose remit is to provide independent advice to the UK Gambling Commission, and is remunerated in this role. He does not provide consultancy to industry bodies. In the past 3 years, he has provided paid consultancy to the Federal Trade Commission (a regulatory agency), the Omidyar Network (a philanthropic investment firm), and PUBLIC (a public sector consultancy). He has been the recipient of an Academic Forum for the Study of Gambling Major Exploratory Grant that is derived from ‘regulatory settlements applied for socially responsible purposes’ received by the UK Gambling Commission and administered by Gambling Research Exchange Ontario (GREO).

## Funding statement

BD is funded by the UK NIHR and UKRI. RG has also received funding from UKRI, the Tech Coalition, and the European Commission. HO is funded by the smart‐data research UK and the European research council. LWP is funded by the NIHR and the Medical Protection Society Foundation. TS has been funded by the Scottish Funding Council. PAT is funded by the NIHR, the UKRI, and the Medical Protection Society Foundation. HW has received grant funding in the last 3 years from UK Research and Innovation Economic and Social Research Council, Wellcome Trust, National Institute for Health Research, Gambling Commission (including regulatory settlements), Office for Health Improvement and Disparities, Greater London Authority, Gambling Research Exchange Ontario, Department of Culture Media and Sport, Trimboos Institute, Blackburn with Darwen Council. DZ is funded by the UKRI.

## Ethics statement

Ethical approval was not required.

## Data Availability

Data sharing is not applicable to this article, as no data sets were generated or analyzed.
